# A bibliometric analysis of cardiotoxicity in cancer radiotherapy

**DOI:** 10.3389/fonc.2024.1362673

**Published:** 2024-04-09

**Authors:** Mengting Che, Yuanqiong Duan, Rutie Yin

**Affiliations:** ^1^Department of Obstetrics and Gynecology, West China Second Hospital of Sichuan University, Chengdu, Sichuan, China; ^2^Key Laboratory of Birth Defects and Related Diseases of Women and Children, Ministry of Education, Sichuan University, Chengdu, China

**Keywords:** radiotherapy, cardiotoxicity, bibliometric analysis, CiteSpace, VOSviewer

## Abstract

**Background:**

Radiotherapy, a primary treatment for malignant cancer, presents significant clinical challenges globally due to its associated adverse effects, especially with the increased survival rates of cancer patients. Radiation induced heart disease (RIHD) significantly impacts the long-term survival and quality of life of cancer survivors as one of the most devastating consequences. Quite a few studies have been conducted on preclinical and clinical trials of RIHD, showing promising success to some extent. However, no researchers have performed a comprehensive bibliometric study so far.

**Objective:**

This study attempts to gain a deeper understanding of the focal points and patterns in RIHD research and to pinpoint prospective new research avenues using bibliometrics.

**Methods:**

The study group obtained related 1554 publications between 1990 and 2023 on the Web of Science Core Collection (WOSCC) through a scientific search query. Visualization tools like CiteSpace and VOSviewer were utilized to realize the visual analysis of countries, authors, journals, references and keywords, identifying the hotspots and frontiers in this research field.

**Results:**

After collecting all the data, a total of 1554 documents were categorized and analyzed using the above tools. The annual number of publications in the field of RIHD shows a continuous growth trend. In 2013, there was a significant rise in the number of linked publications, with the majority of authors being from the USA, according to the statistics. Among all the journals, *INTERNATIONAL JOURNAL OF RADIATION ONCOLOGY BIOLOGY PHYSICS* published the most relevant papers. Cluster analysis of the references showed that research on RIHD has focused on breast cancer, non-small cell lung cancer (NSCLC), and Hodgkin's lymphoma (also among the three main clusters), preclinical research, childhood cancer, heart dose, coronary artery disease, etc, which are also hot topics in the field. High-frequency keywords in the analysis include risk factors, cancer types, heart disease, survival, trials, proton therapy (PT), etc.

**Conclusion:**

Future research on RIHD will mostly focus on thoracic cancer, whose exact cause is yet unknown, with preclinical trials playing an important role. Preventing, consistently monitoring, promptly diagnosing, and timely treating are crucial to decreasing RIHD and extending the life expectancy of cancer survivors.

## 1 Introduction

The incidence of cancer has been rapidly increasing in recent decades, leading to a huge disease burden (1). Radiotherapy is a fundamental component of comprehensive cancer treatment, utilized either independently or as an essential part of the management of various malignant cancer. Radiotherapy is commonly utilized to treat breast cancer, non-small cell lung cancer (NSCLC), Hodgkin’s lymphoma, esophageal cancer, thyroid cancer, prostate cancer, and genital cancer, which greatly improves cancer control and prolongs survival (2, 3). Nevertheless, more survivors are at risk of long-term complications due to radiotherapy, leading doctors to deal with new challenges in managing toxicity that impacts quality of life (4). RIHD was first discovered in the 1970s and research has shown that it can have various effects on the heart, including ischemic heart disease, cardiomyopathy, cardiac dysfunction, and heart failure. These symptoms may worsen significantly years or even decades after exposure to radiation (5). Although radiotherapy has made tremendous progress with techniques like intensity modulated radiotherapy (IMRT), volume modulated arc therapy (VMAT), and PT, off-target cardiotoxicity is unavoidable. It has been realized that RIHD remains a prevalent cause of morbidity and mortality in cancer survivors (6). A large retrospective study found that Hodgkin lymphoma patients who underwent thoracic radiotherapy had a 2.2% higher risk of heart disease morbidity and mortality after 5 years and a 16% higher risk after 20 years compared to patients who did not receive radiotherapy. Similar findings have been observed in other thoracic cancer (7). Researchers are worried that RIHD may offset the benefits of radiotherapy, and moreover, the European Society of Cardiology has demonstrated that RIHD requires further research (8).

Throughout the years, numerous publications, including preclinical trials, clinical trials, systematic reviews, meta-analyses, and practice guidelines, have extensively investigated specific research questions regarding RIHD. However, the abundance of papers increases the difficulty for researchers to stay updated on the newest scientific developments. Reviews can help organize published references and track the progress of an academic field. However, reviews that rely on personal reading and clinical experience may be influenced by subjectivity and lack a comprehensive and broad perspective (9). This problem can be effectively solved with the help of bibliometrics. Compared with traditional reviews, bibliometrics can evaluate information data and depict hotspots and trends in a discipline (10, 11). It is a text mining method based on artificial intelligence algorithms that assesses the interrelationships and impacts of publications and uses mathematical and statistical techniques to identify and organize knowledge structure (12). It not only provides quantitative statistical analysis of publications in a specific field but also helps readers understand the development of the discipline and visualize the frontiers through mapping (13). Bibliometrics plays a crucial role in biomedical disciplines like inflammation, genetics, and cancer, aiding in illness treatment and the development of clinical guidelines (14–16). This study is the first bibliometric review on RIHD. It conducts a graphical analysis of publications, countries, authors, journals, references, and keywords related to RIHD. The study also identifies the hotspots and frontiers of RIHD, with the goal of inspiring clinical and scientific researchers.

## 2 Materials and methods

### 2.1 Data collection

**Figure 1:**
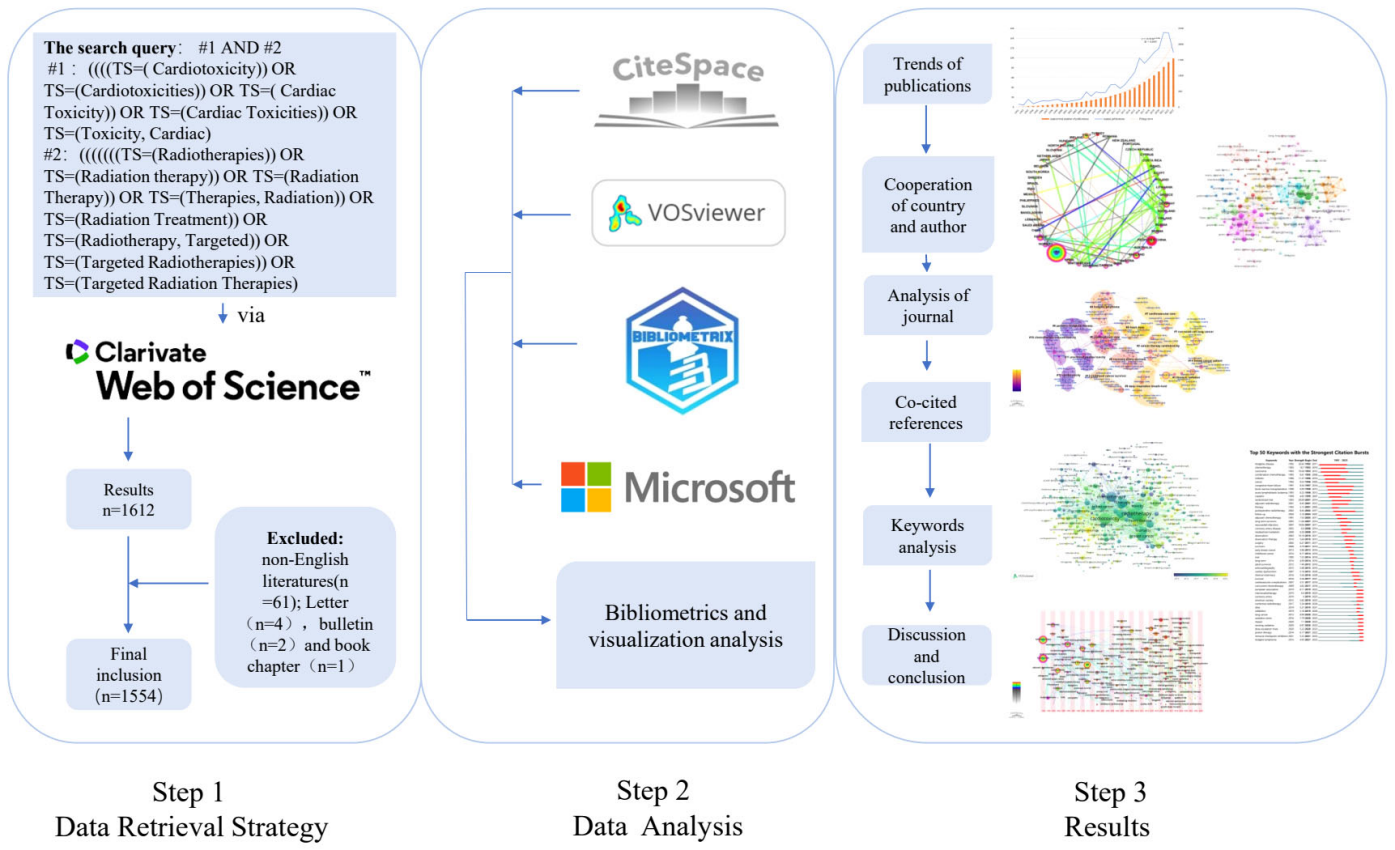
The detailed research process.

The Web of Science Core Collection (WOSCC) is a high-quality, up-to-date, error-free database of more than 12,000 of the most influential and valuable scientific journals. All relevant references have been searched and updated on September 18, 2023, in the Science Citation Index Expanded/SCI-E database in the WOSCC, which is most suitable for bibliometric analysis (17, 18). Radiotherapy (#1) and cardiac toxicity-related (#2) terms are mainly used as descriptors. A Boolean algorithm consisting of “#1 AND #2” is used to ensure that all items retrieved fall within the scope of RIHD. The specific search query is illustrated in **Figure 1**. The study covers research from 1991–2023, with 1612 papers obtained from the WOSCC SCI-E database, including all clinical and preclinical papers. Previous studies have shown that the inclusion of English publications can ensure the accuracy of the analysis (11). Therefore non-English papers are excluded. With the literature types being screened, letters (n = 4), bulletins (n = 2), and book chapters (n = 1) are excluded, which may be non-evidence-based and fallacious (12). Finally, a total of 1,554 publications are included in the final bibliometric and visual analysis. In conclusion, the relevant data in the form of plain text format from WOSCC are exported, including title, author, year of publication, country, institution, keywords, citations, abstracts, and references.

### 2.2 Data analysis and visualization

The study group adopted Microsoft Excel 2020 to draw the annual publication graph and a chart of the top 10 countries in terms of the number of publications, and so on. The group also used bibliometrics analysis software (VOSviewer 1.6.11, CiteSpace 6.2. R4, and Bibliometrix) to analyze and visualize the data of the above 1554 documents.

CiteSpace is a free Java app developed by Professor Chaomei Chen. It detects and visualizes trends and patterns in scientific papers through a progressive knowledge domain visualization methodology that visualizes maps of highly cited and critical documents within knowledge domains (19). CiteSpace produces a number of important metrics. The centrality based on structural void theory measures the number of times a node is located on the shortest path between other nodes. Nodes with higher centrality are considered as critical hubs and play important roles in the information conversion process (20). The clustering method of CiteSpace is spectral clustering, which uses each vertex in the graph as a cluster and merges the clusters by calculating the similarity between different vertices (21). In the clustering analysis, the Q-score measures the extent to which the network can be divided into clusters, and the S-score can verify the internal consistency of data clustering. The closer these two scores are to +1, the more effective the clustering is (22). The burstness demonstrates a specific duration when a sudden change in the frequency of an element occurs, thus identifying emerging terms, which can represent trends and frontiers to some extent (23). In the graphs drawn by CiteSpace, each node represents one element (author, country, institution, or keyword), and the size of the nodes represents the number of publications (author, country, institution) or frequency of occurrence (keyword). In addition, the connecting lines between the nodes represent co-occurrence or co-citation relationships, the thickness of the lines represents the thickness of the correlations, the red nodes represent nodes that are in hot states in the study, and nodes in the purple outer circle have high centrality (centrality > 0.1) (9). With Citespace, the study group drew the cooperation map of the country, the co-cited reference and Keywords time zone diagram, and the references and keywords burst maps to realize the visualization analysis of research status, hotspots, and frontiers.

VOSviewer is a software tool for creating and browsing maps based on web data, primarily for analyzing scholarly records (24). The clustering algorithm on which VOSviewer is based is VOS clustering, which is similar to the Modularity network clustering algorithm (25). In the graphs drawn by VOSviewer, different nodes represent different elements (co-cited references, journals, authors), and the size of the nodes is proportional to the number of publications or the frequency of keywords. Lines between the nodes indicate collaborations or co-citation relationships. Different colored nodes and lines represent different clusters or years (26). With VOSviewer, the study group has implemented a visual analysis of author collaborations and a keywords co-occurrence map.

The study group then used the bibliometric R-package (https://www.bibliometrix.org/home/) to import the data from WOSCC exported to Rstudio software. Then, the impact factor (IF), H-index, and co-citation times of authors and journals in 2023 were obtained. Bibliometrix and Biblioshiny are open-source software packages for use in the R environment. Bibliometrix performs the entire scientific data analysis process, while Biblioshiny enables users to create visualizations based on an interactive web interface (27). Since no patients have participated in this study and the data are all obtained from WOSCC, informed consent and ethical approval are not required. The specific research process is as follows in **Figure 1**.

## 3 Results

### 3.1 Analysis of general information

#### 3.1.1 The publication trend

**Figure 2:**
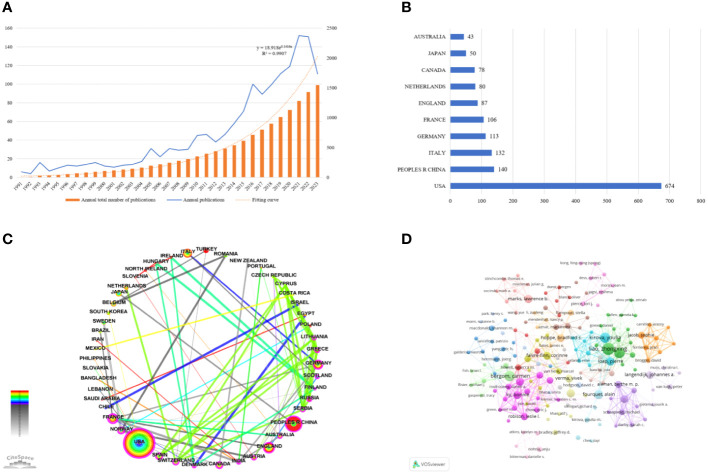
General information. **(A)** The annual publication curve on RIHD. Since the data collection time was September 2023, it is not clear that the number of publications in 2023 is less than that in 2022. **(B)** Chart of the top 10 countries by number of publications. **(C)** Cooperation map of country (nodes=53, links=244).Each node represents a country, and Larger nodes mean more publications. The lines between the nodes represent the cooperative relationship, the thickness of the line represents the thickness of the cooperation relationship. Red nodes mean that the nodes are in hot positions for research, and the purple outer circle represents the high centrality of the node. The image is designed according to centrality. France has the highest centrality, the centrality drops counterclockwise from France (centrality is 0.49). **(D)** Cooperation map of author. Different nodes represent different authors, the size of the nodes is proportional to the number of authors’ literatures, the thickness of the lines between indicates the strength of cooperation, and the clusters of different colors represent the different author cooperation networks.

The number of publications about RIHD has been on the increase from 1991 to 2023. According to the annual publication curve in **Figure 2A**, the annual publication volume can be seen in the early stage (period of 1991–2012) on the slow growth. The rapid growth happens during the period of 2013–2023, as easily shown by the slope of the curve.

#### 3.1.2 Country and author

From 1991 to 2023, a total of 70 countries have participated in the study of RIHD, with the top ten countries being mainly developed countries, as shown in **Figure 2B**. The United States published the most documents with 647 publications. **Figure 2C** shows the cooperation map among countries. Excluding isolated countries, 48 countries are incorporated into the cooperation network map. The top country in centrality is France (0.49), which have established close research cooperation relations with other countries.

Within the timeframe of the study, 8,764 authors have participated in the field. **Figure 2D** shows the cooperation map of authors with more than 4 publications, and 22 clusters are formed, reflecting the small group in the field to some extent. **Table 1** shows the top ten authors of the H-index and their corresponding total cited times, number of publications, and countries from which they come.

**Table 1:** The top ten authors of the H-index and their corresponding total citation times, number of publications and countries.

Rank	Author	H index	Total citations	N	Country
1	Marks LB	15	1741	17	USA
2	Liao ZX	12	615	20	USA
3	Fourquet A	11	1054	19	FRANCE
4	Hudson MM	10	1339	11	USA
5	Langendijk JA	10	373	13	Netherlands
6	Lin SH	10	454	16	USA
7	Simone CB	10	473	15	USA
8	Robison LL	9	1897	11	USA
9	Van Leeuwen FE	9	997	11	Netherlands
10	Aleman BMP	8	951	10	Netherlands

#### 3.1.3 Journal

Worldwide, there are 433 journals that have published documents on RIHD, of which *INTERNATIONAL JOURNAL OF RADIATION ONCOLOGY BIOLOGY PHYSICS* ranks first with 148 literatures. *RADIOTHERAPY AND ONCOLOGY* (n = 69), *JOURNAL OF CLINICAL ONCOLOGY* (n = 42), *CANCER*S (n = 35), and *RADIATION ONCOLOGY* (n = 34) are also important carriers for research reports. *INTERNATIONAL JOURNAL OF RADIATION ONCOLOGY BIOLOGY PHYSICS*, also known as the *red journal* takes the first place in terms of H index(46), total co-citations(6773), and number of publications(n=143), which occupies an authoritative position in the field of RIHD and promotes international cooperation and the advancement of scientific research.

### 3.2 Co-cited reference

**Figure 3:**
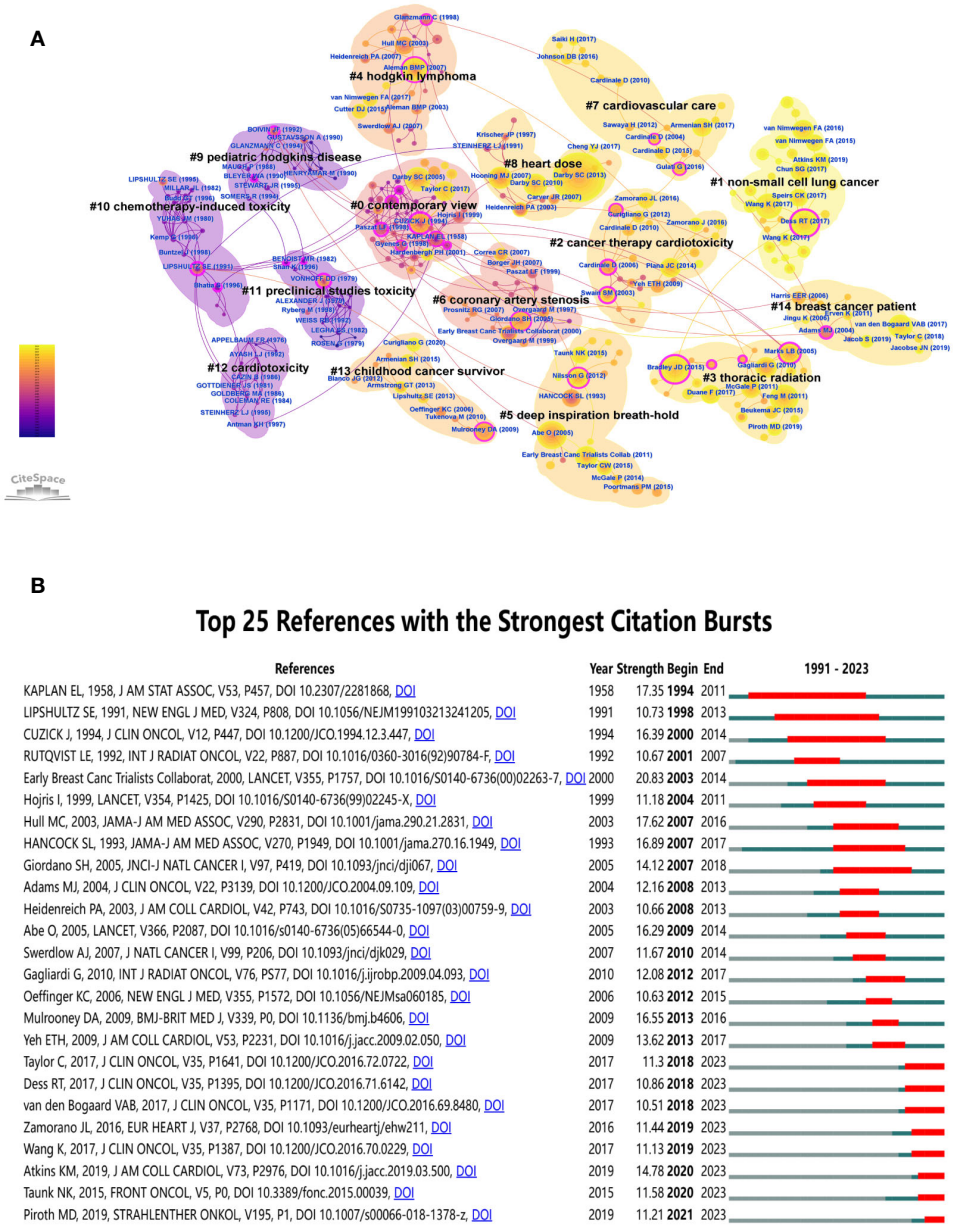
Reference analysis. **(A)** Cluster view of co-cited references in RIHD. **(B)** Top 25 references with the strongest citation bursts. Year represents the year in which the literature was published, strength represents the intensity of emergence (the higher the intensity of emergence, the more researchers pay attention to the literature), and begin and end represent the beginning and end time when the literature is frequently cited, which correspond to the red parts in the figure.

Co-cited references are references cited by more than one publication. Co-cited reference maps are generated with corresponding clusters, which can mention landmark references and research clusters and reflect the trends and frontiers of research in a particular field (28). The retrieved original documents are categorized into 15 clusters using cluster analysis, as shown in **Figure 3A**. Notably, these clusters are well structured (Q = 0.8424) and highly credible (S = 0.9118). It is observed that the publications in each cluster are closely related and coordinated within a particular domain. The cluster labeled “contemporary view” (cluster #0) takes the largest portion, followed by “non-small cell lung cancer” (cluster #1), “cancer therapy cardiotoxicity” (cluster #2), “thoracic radiation” (cluster #3), and “hodgkin lymphoma” (cluster #4). The latest research on RIHD has focused on thoracic cancer, including breast cancer, NSCLC, and Hodgkin’s lymphoma. The purple clusters on the left represent the foundation of the discipline from a certain point of view, including “preclinical studies toxicity (cluster #11), “pediatric Hodgkins disease” (cluster #9), and “cardiotoxicity” (cluster #12). Other important clusters, such as “heart dose” (cluster #8), “breast cancer patient “(cluster #14), “cardiovascular care,” and “childhood cancer survivors” (cluster #13), may represent shifts or breakthroughs in this field.

After sorting out the top 10 cited references on RIHD, it is found that, as shown in **Table 2**, all these documents have been published in high-quality journals. Among them, six publications are related to breast cancer, two are on NSCLC, one is on Hodgkin lymphoma, and one is a review of this field, which fully demonstrates the close relationship and hot position of thoracic radiotherapy, especially breast cancer in this field. Risk of Ischemic Heart Disease in Women After Radiotherapy for Breast Cancer is the most frequently cited reference, in keeping with the fact that the largest node in the co-cited reference map represents Darby SC (2013) (29). The results of this study can often be seen in RIHD-related papers. However, highly cited publications were published earlier than the late, high-quality ones, possibly causing the latter to appear later in low citation. The map of the top 25 references with the strongest citation bursts can make up for this deficiency. As shown in **Figure 3B**, you can see the most recently cited papers. The time cutoff is 2023. They are Piroth MD, 2019 (strength: 11.21, time span: 2021-2023) (30), Taunk NK, 2015 (strength: 11.58, time span: 2020-2023) (31), Atkins KM, 2019 (strength: 14.78, time span: 2020-2023) (32), etc.

**Table 2:** The top ten cited publications and their corresponding cited times, the author and publication year of the publication and published journal.

Rank	Title	Total Citations	Author, Year	Journal (JCR partition, IF)
1	Risk of ischemic heart disease in women after radiotherapy for breast cancer	395	Darby SC, 2013	*NEW ENGL J MED*, (Q1, 158.5)
2	Effects of radiotherapy and of differences in the extent of surgery for early breast cancer on local recurrence and 15-year survival: an overview of the randomized trials	175	Abe O, 2005	*LANCET* (Q1, 168.9)
3	Development and validation of a heart atlas to study cardiac exposure to radiation following treatment for breast cancer	137	Feng M, 2011	*INT J RADIAT ONCOL* (Q1, 7)
4	Long-term mortality from heart disease and lung cancer after radiotherapy for early breast cancer: prospective cohort study of about 300,000 women in US SEER cancer registries	134	Darby SC, 2005	*LANCET ONCOL* (Q1, 51.1)
5	Standard-dose versus high-dose conformal radiotherapy with concurrent and consolidation carboplatin plus paclitaxel with or without cetuximab for patients with stage IIIA or IIIB non-small-cell lung cancer (RTOG 0617): a randomized, two-by-two factorial phase 3 study	117	Bradley JD, 2015	*LANCET ONCOL* (Q1, 51.1)
6	Cardiac Toxicity After Radiotherapy for Stage III Non-Small-Cell Lung Cancer: Pooled Analysis of Dose-Escalation Trials Delivering 70 to 90 Gy	113	Wang K, 2017	*J CLIN ONCOL* (Q1, 45.3)
7	Late cardiotoxicity after treatment for Hodgkin lymphoma	108	Aleman BMP, 2007	*BLOOD* (Q1,20.3)
8	Radiation-related heart disease: current knowledge and future prospects	105	Darby SC, 2010	*INT J RADIAT ONCOL* (Q1, 7)
9	Cause-specific mortality in long-term survivors of breast cancer who participated in trials of radiotherapy.	103	CUZICK J, 1994	*J CLIN ONCOL* (Q1, 45.3)
10	The incidence and functional consequences of RT-associated cardiac perfusion defects	102	Marks LB, 2005	*INT J RADIAT ONCOL* (Q1, 7)

### 3.3 Keyword

#### 3.3.1 Keywords occurrence analysis

**Figure 4:**
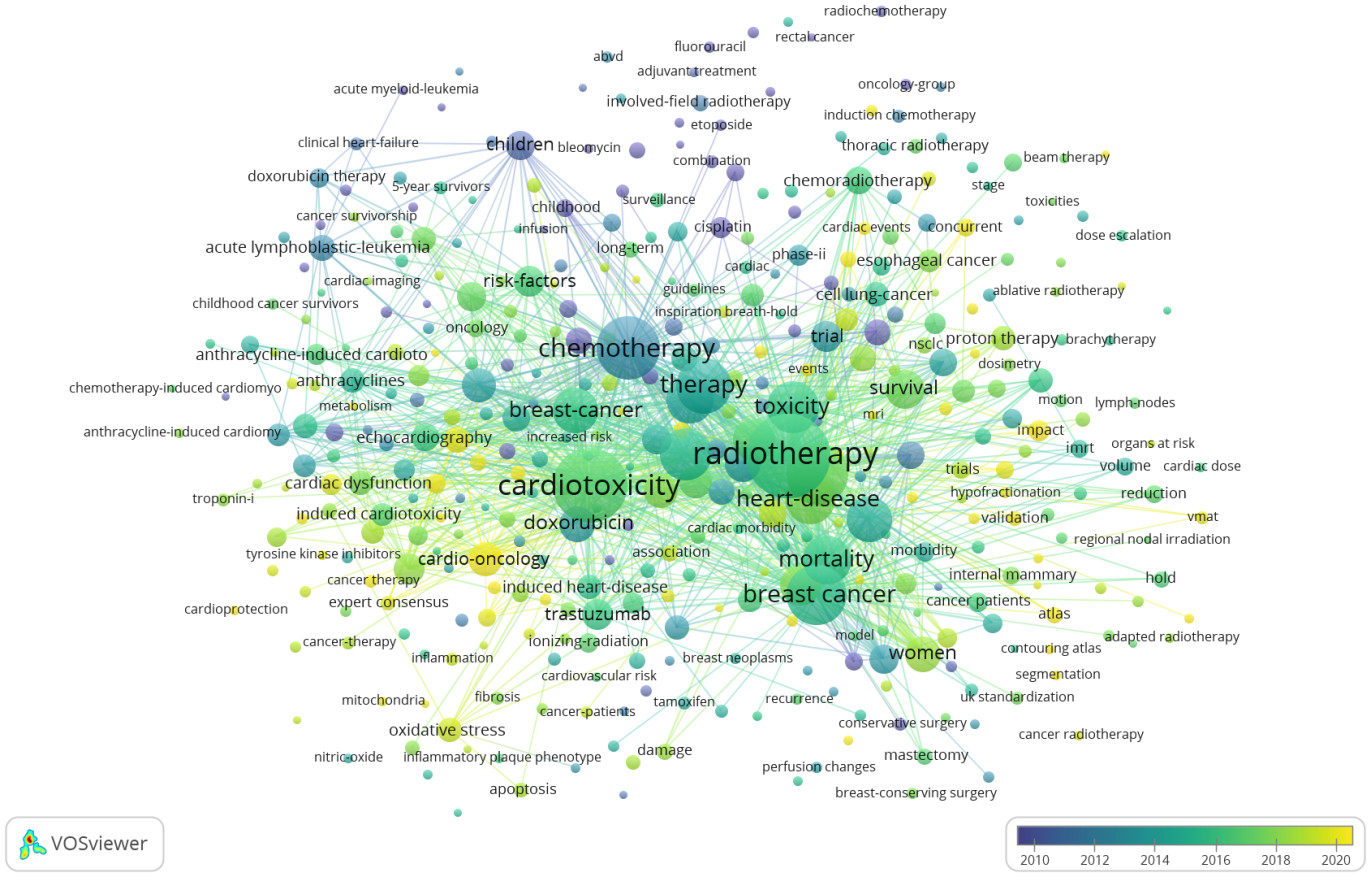
Keywords occurrence map. Different nodes represent different keywords, the size of the nodes is proportional to the occurrence times of the keywords, the thickness of the lines between the nodes the co-occurrence strength of the keywords.

Keywords reflect the focus issue and summary content of the document, and the research hotspots in the field can be seen from the frequency of keywords (33). VOSviewer excels at creating, visualizing, and exploring keywords co-occurrence maps (26). The keywords occurrence map drawn by VOSviewer can be seen in **Figure 4**. The most frequently occurring keywords include radiotherapy (n = 525), cardiotoxicity (n = 407), risk (n = 280), breast cancer (n = 263), chemotherapy (n = 291), heart disease (n = 195), mortality (n = 184), women (n = 99), survival (n = 112), cardio-oncology (n = 81), childhood cancer (n = 64), coronary artery disease (n = 60), trial (n = 76), lung cancer (n = 59), American society (n = 52), Hodgkins disease (n = 52), management (n = 53), proton therapy (n = 46), intensify-modulated radiotherapy (n = 48), conformal radiotherapy (n = 44), etc, which are the hotspots of current research and represent the focus of scholars in the past 30 years. The yellow nodes represent the latest keywords, including cardio-oncology, oxidative stress, atlas, expert consensus, MRI, etc, which can represent the frontiers of disciplinary development to some extent.

#### 3.3.2 Keywords time zone diagram

**Figure 5:**
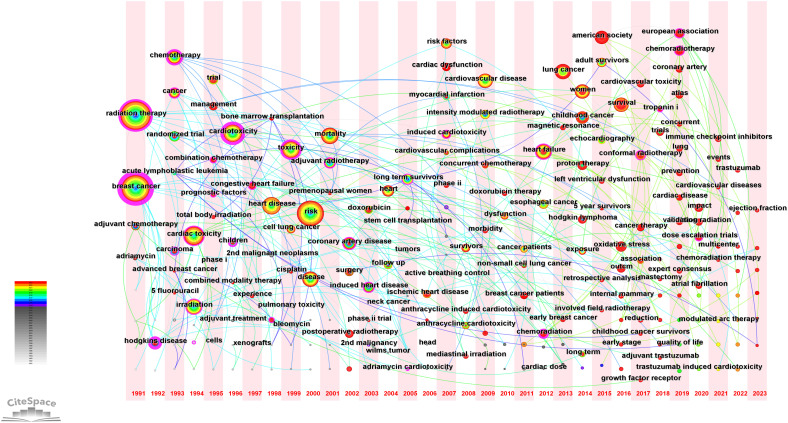
Keyword time zone diagram. It describes the year when keywords first appeared in the data set, and each time period presents all newly emerged keywords in that time period, showing the evolution relationship of keywords.

With CiteSpace, the study group drew the map of Keywords time zone diagram (**Figure 5**), which shows the evolution relationship of keywords. A closer look reveals that we can divide the development of RIHD from 1990-2023 into three phases. The first phase is the initial awareness phase (1990-2000), the keywords of which mainly include radiotherapy, cardiotoxicity, cancer types (breast cancer, hodgkins disease and cell lung cancer), chemotherapy (adriamycin and 5 fluorouracil), children, trial, etc. Initial awareness of the close relationship between RIHD and thoracic cancer and the close association of chemotherapy and age with cardiotoxicity after cancer treatment led to a series of preclinical and clinical studies in these three major cancer types. The second phase (2000-2010) can be summarized as the phase of risk analysis, the awareness of cardiac events corresponding to RIHD, and some initial exploration of methods to reduce RIHD-related deaths and increase survival. The keywords in this section are mainly risk factors, mortality, survival, cardiovascular disease, intensify modulated radiotherapy, active breath holding, etc. The third phase (2010-2023) can be summarized as the latest exploration of the mechanisms and means of prevention and treatment of RIHD. Keywords of this part manly include oxidative stress, magnetic resonance, atlas, proton therapy, conformal radiotherapy, modulated arc therapy, growth factor receptor, troponin i, expert consensus, American society, etc. At this stage, the focus of researchers is on the micro-level mechanisms of RIHD, which needs to be supported by a large number of preclinical studies. Researchers aim to use advanced technologies or molecular markers to detect RIHD at an early stage, and to treat or even prevent RIHD. Experts and organizations around the world are collaborating to do so, meaning that RIHD is a global challenge.

#### 3.3.3 Keywords burst map

**Figure 6:**
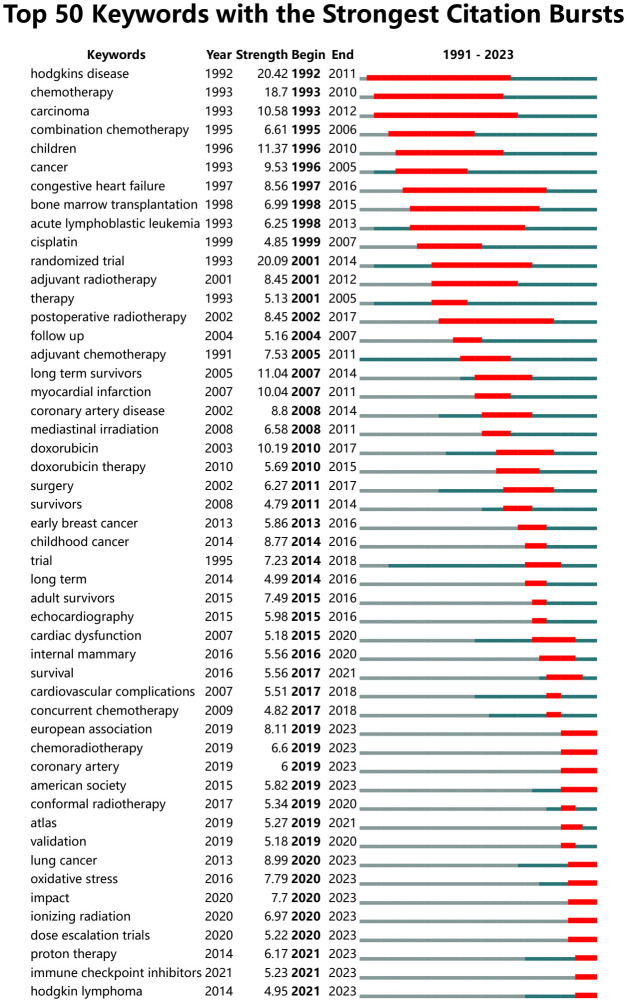
Top 50 keywords with the strongest citation burst. Refer to **Figure 3B** for the meaning of each part of the diagram.

Emergent words that appear or are cited frequently in a certain period of time can help researchers analyze the evolution of RIHD research as well as highlight emerging topics (34). The map of the top 50 keywords with the strongest citation burst (**Figure 6**) is obtained via CiteSpace. This graph is largely consistent with what is depicted in the time zone diagram above. European Association (strength, 8.11;time span, 2019-2023), chemoradiotherapy (strength, 6.6;time span, 2019-2023), coronary artery (strength, 6;time span, 2019-2023) oxidative stress (strength, 7.79; time span, 2020-2023), ionizing radiation (strength, 6.97;time span, 2020-2023), dose escalation trials (strength, 5.22;time span, 2020-2023), proton therapy (strength, 6.17;time span, 2021-2023), immune check point inhibitors (strength, 5.23;time span, 2020-2023) are the research hotspots in the decade, indicating the latest research focuses on the molecular mechanism of cardiotoxicity and the radiological technology. Radiation to cardiac substructures has recently received more attention, and the use of immune checkpoint inhibitors in conjunction with radiotherapy for cancer treatment has also been a recent hot topic of research. And the European Association is the most popular research society at present. These topics are likely to repeat in the near future.

## 4 Discussion

### 4.1 General information

This study demonstrates the publishing level in the field of RIHD, the collaboration among countries, authors, and journals, as well as the present research focus and trends through bibliometrics and visualization analysis. From 1991 to 2023, a scientific search yielded 1554 relevant works authored by 8764 individuals from 70 countries and published in 433 academic journals.

Prior to the year of the first analysis paper in this study, only 2 relevant documents were retrieved from WOSCC. The paper published in 1979 indicated that radiotherapy might enhance the cardiotoxicity of adriamycin in patients with stage IV breast cancer, and the other published in 1981 made the initial attempt to describe cardiotoxicity after chemotherapy and radiotherapy (35, 36). The annual publication volume serves as a significant metric for assessing the robustness and expansion of RIHD during a specific time frame. Prior to 2013, the annual output had increased gradually but consistently, with a general upward trend. The study conducted by Darby SC in 2013 subsequently accelerated the rate of subsequent annual publications. It is the most cited publication and is regarded as a significant advance in RIHD research. A novel linear correlation between radiation dose and cardiotoxicity was established in this nearly 50-year-long multicenter cohort study conducted in Sweden and Denmark: the mean heart dose (MHD) was found to be linearly correlated with the incidence of major coronary events in breast cancer patients. The increase in incidence was 7.4% per gray (95% confidence interval: 2.9% to 14.5%; P<0.001). Cardiac events manifested as long-term effects and women with cardiac risk factors had a higher risk of developing RIHD after radiation. These findings served as the foundation for subsequent investigations (29). Furthermore, it is worth noting that around 71% of the extracted publications were published subsequent to 2013, indicating that RIHD has gathered a growing level of scholarly attention in recent times. The analysis of country and author shows that the United States has contributed the largest number of publications and the largest number of high-quality authors and institutions, with Marks LB being the author with the highest H-index. Notwithstanding its dominant position, the United States does not possess a “monopoly” in this field. China, the only developing nation to rank among the top ten nations in terms of publications, is presently ranked second in terms of the greatest quantity of RIHD research outputs. While Italy may not have produced the most publications, it has demonstrated the most intimate collaboration with other nations that have the highest centrality. Countries and authors have collectively established an intimate network of collaboration and endeavored to advance the progress of this domain, thereby unequivocally exposing RIHD as a worldwide predicament. The most productive and influential journal is *INTERNATIONAL JOURNAL OF RADIATION ONCOLOGY BIOLOGY PHYSICS*. By diligently monitoring these authors, and journals, researchers can remain informed about the most recent developments in the respective field.

### 4.2 Knowledge base

Over the past 40 years, research has increased our understanding of the mechanism of RIHD. Acute and persistent inflammation, endothelial dysfunction, and reactive oxygen species (ROS) all contribute to chronic fibrosis of the myocardium, which is a critical component of RIHD (37). Oxidative stress is the most recent area of this field. Radiation breaks down the respiratory chain of mitochondrial metabolism in old endothelial cells. This makes mitochondria more permeable and creates a lot of ROS (1). When ROS exceeds the capacity of intracellular antioxidants, large molecules such as DNA, proteins, and lipids are damaged. Moreover, ROS can initiate crucial mechanisms that result in apoptosis and necrosis, including mitochondrial permeability transition and calcium release. Excess ROS mediates a range of molecular signaling pathways, promoting the release of related cell factors leading to inflammation and fibrosis in RIHD, such as tumor necrosis factor, transforming growth factor-β, IL-4, nuclear factor-kappa β, etc (38, 39).

Research on RIHD has primarily been carried out in animal models, contributing to the understanding of the biological pathways and mechanisms involved in normal tissue toxicity in radiotherapy, identifying potential therapeutic targets at the cellular and molecular levels, and translating to clinical studies to better determine radiation dose (40). Currently, there is a trend to evolve from whole thorax to whole heart to partial heart irradiation in animal models used for RIHD (41). Whole thorax irradiation is a method that has been widely used in the past and does not require precise image guidance during radiation (42). However, its radiation field includes both the lungs and the heart, making it difficult to determine that the damage is solely from cardiac irradiation (43). Whole heart irradiation is a relatively new technique that requires imaging to accurately deliver radiation to the heart using a beam size that is focused primarily on the heart, thus limiting the lung dose (44). MHD is an important measure for evaluating the level of radiation exposure to the heart in cardiac procedure (29). However, more and more studies suggest that there is a closer relationship between RIHD and cardiac substructures receiving high dose of radiation, among which the dose of coronary artery is considered to be an independent factor in the assessment of RIHD and has a strong association with risk of ischemic heart disease (45). Therefore, there is a need to enhance preclinical models of the substructures of the heart, including key components such as the coronary artery, in order to accurately predict risk factors and successfully develop interventions for RIHD. Radiotherapy is frequently used alongside surgery, chemotherapy, hormones, and immunotherapy in cancer treatment, all of which may lead to cardiotoxicity (42, 46, 47). As immune checkpoint inhibitors are increasingly used in combination with radiotherapy in the treatment of NSCIL, the cardiotoxicity associated with them, which is relatively rare but has a high mortality rate, is of concern. Chemotherapy is an independent risk factor for cardiotoxicity, with medicines like doxorubicin, 5-fluorouracil, and anthracycline being recognized for causing cardiotoxicity (47). An analysis of the SEER database revealed a notable rise in the occurrence of cardiac events in patients who received a substantial cumulative dosage of anthracyclines along with adjuvant radiotherapy (RR: 1.26; 95% CI: 1.12-1.42) (48). A study using a New Zealand white rabbit model showed that complete cardiac irradiation plus adriamycin had a synergistic impact on cardiotoxicity (49). Animal models can be utilized to investigate the interactions between various medicines and their impact on short and long-term cardiovascular outcomes, as well as to enhance the timing and order of combined treatments. Preclinical animal models of mice, rats, rabbits, dogs, pigs, and nonhuman primates are often used in RIHD research (50). The rodent is a valuable study model that provides insights into many proteins and processes related to RIHD. The rabbit shares similarities with humans in cardiovascular physiology, particularly in ion channel and calcium transporter function (51). In pigs, the coronary circulation is comparable to that of young individuals, but in dogs, it resembles that of older individuals with ischemic heart disease (52, 53). Yet, rodents are restricted by their substantial anatomical disparities from humans, whereas rabbits, dogs, and primates are restricted by financial and moral considerations. Additionally, factors such as animal age, size, and sex must be meticulously taken into account when choosing models, as they can significantly impact experimental outcomes (41, 50).

### 4.3 Hotspots and frontiers

Analysis of keywords shows that RIHD-associated cancer types are mainly referred to thoracic cancer, including breast cancer, NSCLC and Hodgkin’s lymphoma, which most of the latest trials are based on (4). Many publications on RIHD are based on breast cancer and have resulted in numerous established findings in the field. As the majority of breast cancer patients are female, “women” is a common keyword. The linear relationship between cardiotoxicity and radiation dose was first put forward in breast cancer and a similar linear relationship was also observed in Hodgkin’s lymphoma (29, 54). The RIHD of breast cancer has a significant laterality, which means a significantly increased risk of cardiovascular death after radiation in patients with left-sided breast cancer happens (55). It was previously thought that heart exposure to radiation was not important in NSCLC due to the short survival time and the well-known risks of pneumonia and esophagitis. But recent investigations have increasingly cast doubt on this perspective. The 74GY group in the randomized, phase III study of RTOG0617 showed significantly poorer overall survival compared to the 60GY group, with 20.3 months against 28.7 months, respectively (p = 0.004). Researchers analyzed several factors and found that the cardiac dose of the 74GY group was significantly higher than that of the 60GY group, which likely accounts for this phenomenon (56). Available studies suggest that NSCLC patients may experience RIHD up to two years after radiation exposure (57). Hodgkin’s lymphoma is common in young people and typically has a favorable prognosis, leading to survivors being consistently at risk of RIHD. Hodgkin’s lymphoma exemplifies change in radiotherapy plan. In order to minimize cardiac radiation, Hodgkin’s lymphoma radiation ranges from the entire field to the affected nodules, with the dose decreased from 44 GY to 20 GY (58, 59). Radiotherapy in conjunction with chemotherapy is now the predominant therapeutic approach for Hodgkin’s lymphoma, leading to a significant decrease in the occurrence of cardiac events (60).

Research on the risk factors associated with RIHD has consistently been the primary emphasis, which can be categorized as radiation-related or patient-related. Risk factors associated with radiation include total radiation dosage, average cardiac dose, primary cancer location, heart volume exposed to radiation, and whether shielding is used during therapy (3). MHD is a crucial sign for evaluating cardiac exposure. Recent studies indicate that targeting radiation to specific cardiac substructures is better predictive of cardiotoxicity (45). Further research is required to determine the most accurate metrics for predicting cardiotoxicity. RIHD can be influenced by inherent risk factors in patients, such as traditional cardiovascular risk factors (age >65 years, diabetes mellitus, smoking, and obesity), high blood pressure, sedentary lifestyle, history of cardiovascular disease, and young age at the time of radiotherapy (61). The incidence of RIHD is higher than that of cancer survivors without these risk factors, as demonstrated by a high-quality cohort Dutch study (62). RIHD following radiation at a young age has distinct characteristics among these risk factors. Thanks to modern medical advances, the long-term survival rate for childhood cancer has improved dramatically. However, the risk for RIHD is higher in children from the onset of radiation because there is more time for classical cardiovascular risk factors to act simultaneously on children (63). Moreover, children’s underdeveloped cardiovascular tissue may be more vulnerable to radiation exposure (62). Mulrooney and his colleagues conducted a study on 14,358 children who underwent radiation between 1970 and 1986. They compared these youngsters with their siblings to investigate the risk of developing heart disease later in life. The cumulative incidence of RIHD increased by 5–6 times compared to their siblings over time (64). Cardiovascular events are the primary cause of non-cancer mortality in childhood cancer survivors, indicating a need for additional study in this population.

Cardiovascular events after radiotherapy mainly include coronary artery disease, valvular disease, cardiomyopathy, pericardial disease, and conduction system abnormalities. These cases differ in duration and characteristics but typically result in heart failure and mortality (5). Coronary artery disease is the most prevalent kind of heart damage. The condition may manifest as early symptoms of acute coronary syndrome even sudden cardiac death, but typically occurs around 15 years following exposure to radiation (65). The lesions occur mainly at the opening or proximal part of the coronary arteries, especially the left anterior descending branch of the heart, which is highly susceptible to radiation (66). Imaging tools such as echocardiography, cardiovascular computed tomography, cardiovascular magnetic resonance (CMR), and nuclear cardiology aid in the early detection of cardiac events. Each of these tools has its specific advantages (3). Echocardiography is the predominant and initial imaging technique due to its low risk and easy accessibility. This method is commonly utilized to evaluate the heart’s systolic and diastolic functioning and is more effective in identifying valvular and pericardial disorders (63). Cardiovascular computed tomography is particularly advantageous for detecting coronary stenosis and determining the amount of calcium in the coronary arteries, as well as the presence of soft plaque burdens (67). CMR is quite accurate in detecting and diagnosing ischemic heart disease (68). Using the serum marker is a promising method for identifying and tracking RIHD in clinical practice. Currently, the relationship between conventional cardiac markers such as troponin T, BNP, and NT-pro BNP and radiation is uncertain (69). Markers of vascular endothelial cell inflammation such as placental growth factor (PlGF), tumor necrosis factor α, IL-2, IL-6, and IL-10 have been observed to rise in mice models following radiation in preclinical studies. Notably, there is a strong correlation between PlGF levels and MHD, with a correlation coefficient of 0.89 (70). Additional preclinical and clinical trials are required to confirm their effectiveness (NCT04305613, NCT03978377).

To enhance the long-term cardiac outcomes of cancer survivors, it is crucial to use technologies rationally to identify at-risk individuals before and after therapy, ensuring reliable and early detection of anomalies and prompting intervention to prevent poor outcomes (71). Preventing traditional cardiovascular risk factors is beneficial, including educating patients about maintaining a healthy lifestyle, quitting smoking, and lowering obesity, hypertension, and diabetes. An investigation by SEER-Medicare on breast cancer patients found that the absolute risk of cardiac death in the left breast decreased from 13% (1973–1979) to 9.5% (1980–1984) to 5.8% (1985–1989), possibly due to improvements in radiation dose reduction (72). Some ways for reducing radiation dose have been explored. Several straightforward physical techniques, including deep inspiratory breath-holding (DIBH), breathing gating, and posture change, can effectively decrease the cardiac radiation dosage (73–75). DIBH requires patients to take deep breaths during treatment to shift the heart away from the front chest wall and the expanded lung tissue in the middle to reduce the cardiac dose. MHD and the dose to the anterior descending branch of the left coronary artery both decrease notably. However, patients should be instructed carefully to enhance their compliance (73). Progress in radiation technology has been crucial in decreasing radiation exposure to the heart. Advances in radiotherapy techniques, from 2D to 3D conformal radiation therapy (3DCRT) to rotational methods like IMRT and VMAT to PT, have significantly decreased radiation exposure to nearby organs at risk and expanded the treatments’ capability to encompass intricate anatomical structures (3, 76–78). Prior to the advent of CT in the 1980s, radiation therapy used 2D radiographic film planning, making it challenging to prevent and measure heart radiation. Following the extensive utilization of CT, 3DCRT allowed for quantification of the cardiac dose, but cardiac avoidance often resulted in underdose to the target area. IMRT and VMAT, developed since the 1990s, were able to better modulate the dose from each beam through multileaf collimation thus allowing for a more conformal delivery of radiation (79). IMRT has become a standard of care radiotherapy technique for NSCLC and is also widely used to treat patients with left-sided breast cancer (80). A study on left-sided breast cancer patients who had radiotherapy found that the VMAT group had a considerably lower coronary heart dosage compared to the 3DCRT group (81). PT is the latest treatment that uses proton particles instead of traditional X-rays to treat cancer targets, keeping the radiation dose to the heart at a low level and enhance the lethality of cancer cells (78). PT uses the Bragg peak phenomenon to deliver the highest dose at a precise tissue depth, ensuring the tumor receives the intended dose while sparing normal tissue from exposure, hence reducing toxicity to healthy tissue (82). In a phase IIB randomized controlled trial, patients with locally advanced esophageal cancer were compared PT and IMRT. The study revealed that patients treated with PT had a significantly lower MHD compared to those in the IMRT group (19.8 vs. 11.3 GY; P < 0.001). During the follow-up period, the PT group experienced fewer cardiac incidents compared to the IMRT group (83). PT has gained more attention recently for reducing cardiac radiation dose and has been studied in several malignancies, including breast cancer and Hodgkin’s lymphoma. As RIHD is closely linked to the amount of radiation the heart receives, it may be deduced that the decrease in cardiac radiation dose from PT helps reduce RIHD. Nevertheless, assessing the therapeutic impact of this may necessitate an extended follow-up period, as indicated by the following clinical trial registrations: NCT02603341, NCT04361240, NCT04305613, and NCT01993810. Aspirin, statins, and colchicine, which are anti-inflammatory medications, have been commonly used to avoid negative cardiovascular events, and some preclinical research indicates that these treatments may also help prevent RIHD (5, 84). However, there is little clinical trial data on these radiation mitigants, and there are no existing guidelines on the use of anti-inflammatory medications for the prevention of RIHD. Anti-inflammatory drugs show tremendous potential in the field of RIHD (85).

### 4.4 Future and challenges

Radiotherapy techniques have improved considerably over the past 40 years, and the radiation dose is well controlled, so the credibility of old research data has been doubted. The probability of RIHD increases with increasing radiation dose and guidelines state that limiting MHD is a benefit for cancer patients (86). However, as radiation technology evolves in a more conformal direction, such as IMRT and PT, some studies have shown the radiation dose to substructures is a better predictor of the risk of developing RIHD than MHD, so is it more beneficial for researchers to limit the radiation dose to substructures? (45). Animal models aid in the study of heart substructure, and various institutions have developed cardiac contour atlases to aid in substructure delineation. Several cardiac contour atlases have been established and developed, such as Duane, Feng, and Kong’s atlases (87–89). Preclinical trials and animal models also help to elucidate the pathways and mechanisms of RIHD and explore potential therapeutic targets. Anti-inflammatory drugs such as statins, aspirin, and colchicine may be beneficial in preventing and mitigating RIHD, and some animal models have explored the mechanisms involved, but more preclinical trials and high-quality clinical trials are required to back it up (85). Multimodal therapies such as surgery plus chemotherapy and radiotherapy are recognized as cancer treatments, and it needs to reconsider the potential synergistic cardiac-related adverse events where animal models can play a huge role (41). However, the patient situation in the real world is complex and varied, and so far there is no preclinical model that can perfectly reproduce the complex physiopathology of RIHD. The development of a reliable and effective preclinical model that better simulates the real-world situation is particularly important (90). The serum marker can reflect RIHD to a certain extent, but the mechanism and validity of these markers cannot be determined. Some prospective studies are underway (69, 70). More high-quality prospective studies examining advanced radiotherapy techniques such as PT and long-term monitoring of follow-up data are needed to understand whether these advanced techniques can ensure the efficacy of radiotherapy while reducing RIHD and mortality, as strict cardiac dose restriction may compromise the effectiveness of radiotherapy in the treatment of cancer patients (91). In order to optimize the long-term survival rate of cancer patients with RIHD, the department of cardiac-oncology has been established, and oncology and cardiology organizations around the world have fully recognized the importance of collaboration. Through a global multidisciplinary collaboration, multiple expert consensus guidelines have been published for RIHD limitation strategies based on available data and cancer types. Relevant people should be familiar with these guidelines for screening and mitigation of RIHD in adults and children, which advocate cardiovascular risk assessment and reduction before and after radiotherapy, as well as cardiovascular imaging at appropriate follow-up intervals for early identification of subclinical cardiovascular disease and for the management of RIHD throughout its course (92–94).

## 5 Limitation

The data in this study are from the WOSCC SCIE database, without containing all the literature in the field, so the results cannot represent all the current research status of RIHD for cancer. In addition, because the co-citation frequency is related to time, high-quality literature in recent years may have a low co-citation frequency due to a short publication time, which is biased from the actual situation. When using VOSviewer and CiteSpace for data visualization analysis, there is no standard reference for data time partitioning, threshold value, or cropping method, which may cause bias.

## 6 Conclusion

RIHD has gathered significant interest from researchers recently and ranks among one of the primary causes of death in cancer patients following radiation. This study represents the initial bibliometric analysis of RIHD, serving as a guide for future research. Preclinical studies using animal models are crucial for identifying potential targets of drug action, understanding the mechanisms of RIHD and radiation mitigants, and reducing radiation dose. Consistent surveillance, prompt detection, timely medical care, and preventive measures are significant in the intervention of RIHD. Thus, high-quality trials on serum markers of RIHD, imaging methods, radiation mitigants, and advanced radiotherapy techniques are critical. As the field of cardio-oncology continues to make progress in identifying the epidemiology and mechanisms of RIHD, continuously updated cardioprotective strategies are needed to ultimately achieve the goal of RIHD minimization.

## Author contributions

MC: Writing – original draft, Writing – review & editing, Conceptualization, Data curation, Formal analysis, Investigation, Methodology, Software. RY: Writing – review & editing, Funding acquisition, Project administration, Resources, Supervision. YD: Data curation, Writing – review & editing.

## References

[1] Wei T Cheng Y . The cardiac toxicity of radiotherapy - a review of characteristics, mechanisms, diagnosis, and prevention. Int J Radiat Biol. (2021) 97:1333–40. doi: 10.1080/09553002.2021.1956007 34264176

[2] Lee MS Finch W Mahmud E . Cardiovascular complications of radiotherapy. Am J Cardiol. (2013) 112:1688–96. doi: 10.1016/j.amjcard.2013.07.031 24012026

[3] Podlesnikar T Berlot B Dolenc J Goričar K Marinko T . Radiotherapy-induced cardiotoxicity: the role of multimodality cardiovascular imaging. Front Cardiovasc Med. (2022) 9:887705. doi: 10.3389/fcvm.2022.887705 35966531 PMC9366112

[4] Badiyan SN Puckett LL Vlacich G Schiffer W Pedersen LN Mitchell JD . Radiation-induced cardiovascular toxicities. Curr Treat Options Oncol. (2022) 23:1388–404. doi: 10.1007/s11864-022-01012-9 PMC13234928 36087234

[5] Kirova Y Tallet A Aznar MC Loap P Bouali A Bourgier C . Radio-induced cardiotoxicity: From physiopathology and risk factors to adaptation of radiotherapy treatment planning and recommended cardiac follow-up. Cancer Radiother. (2020) 24:576–85. doi: 10.1016/j.canrad.2020.07.001 32830054

[6] Koutroumpakis E Palaskas NL Lin SH Abe JI Liao Z Banchs J . Modern radiotherapy and risk of cardiotoxicity. Chemotherapy. (2020) 65:65–76. doi: 10.1159/000510573 33049738

[7] Galper SL Yu JB Mauch PM Strasser JF Silver B Lacasce A . Clinically significant cardiac disease in patients with Hodgkin lymphoma treated with mediastinal irradiation. Blood. (2011) 117:412–8. doi: 10.1182/blood-2010-06-291328 20858859

[8] Zamorano JL Lancellotti P Rodriguez Muñoz D Aboyans V Asteggiano R Galderisi M . 2016 ESC Position Paper on cancer treatments and cardiovascular toxicity developed under the auspices of the ESC Committee for Practice Guidelines: The Task Force for cancer treatments and cardiovascular toxicity of the European Society of Cardiology (ESC). Eur Heart J. (2016) 37:2768–801. doi: 10.1093/eurheartj/ehw211 27567406

[9] Sabe M Pillinger T Kaiser S Chen C Taipale H Tanskanen A . Half a century of research on antipsychotics and schizophrenia: A scientometric study of hotspots, nodes, bursts, and trends. Neurosci Biobehav Rev. (2022) 136:104608. doi: 10.1016/j.neubiorev.2022.104608 35303594

[10] Chen C . Searching for intellectual turning points: progressive knowledge domain visualization. Proc Natl Acad Sci USA. (2004) 101:5303–10. doi: 10.1073/pnas.0307513100 PMC387312 14724295

[11] Wallin JA . Bibliometric methods: pitfalls and possibilities. Basic Clin Pharmacol Toxicol. (2005) 97:261–75. doi: 10.1111/j.1742-7843.2005.pto_139.x 16236137

[12] Guler AT Waaijer CJ Palmblad M . Scientific workflows for bibliometrics. Scientometrics. (2016) 107:385–98. doi: 10.1007/s11192-016-1885-6 PMC4833826 27122644

[13] Duan Y Zhang P Zhang T Zhou L Yin R . Characterization of global research trends and prospects on platinum-resistant ovarian cancer: a bibliometric analysis. Front Oncol. (2023) 13:1151871. doi: 10.3389/fonc.2023.1151871 37342181 PMC10277726

[14] Wang Z Huang C Li X . Research trends and hotspot analysis of conjunctival bacteria based on CiteSpace software. BioMed Res Int. (2020) 2020:2580795. doi: 10.1155/2020/2580795 33083458 PMC7556104

[15] Ahn SK Hwang JW . Global trends in immunotherapy research on breast cancer over the past 10 years. J Oncol. (2020) 2020:4708394. doi: 10.1155/2020/4708394 33204263 PMC7661143

[16] Avcu G Sahbudak Bal Z Duyu M Akkus E Karapinar B Vardar F . Thanks to trauma: A delayed diagnosis of Pott disease. Pediatr Emerg Care. (2015) 31:e17–8. doi: 10.1097/PEC.0000000000000637 26626903

[17] Aggarwal A Lewison G Idir S Peters M Aldige C Boerckel W . The state of lung cancer research: A global analysis. J Thorac Oncol. (2016) 11:1040–50. doi: 10.1016/j.jtho.2016.03.010 27013405

[18] Jiang M Qi Y Liu H Chen Y . The role of nanomaterials and nanotechnologies in wastewater treatment: a bibliometric analysis. Nanoscale Res Let. (2018) 13:233. doi: 10.1186/s11671-018-2649-4 30097816 PMC6086776

[19] Synnestvedt MB Chen C Holmes JH . CiteSpace II: visualization and knowledge discovery in bibliographic databases. AMIA Annu Symp Proc. (2005) 2005:724–8. PMC1560567 16779135

[20] Freeman LC . A set of measures of centrality based on betweenness. Sociometry. (1977) 40:35–41. doi: 10.2307/3033543

[21] Brandes U . Mapping knowledge domains of industrial structure research in China: 1992-2015. J Discret Math Sci C. (2017) 20:1393–7. doi: 10.1080/09720529.2017.1392452

[22] Shibata N Kajikawa Y Takeda Y Matsushima K . Detecting emerging research fronts based on topological measures in citation networks of scientific publications. Technovation. (2008) 28:758–75. doi: 10.1016/j.technovation.2008.03.009

[23] Kleinberg J . Bursty and hierarchical structure in streams. Data Min Knowl Discovery. (2003) 7:373–97. doi: 10.1023/A:1024940629314

[24] Arruda H Silva ER Lessa M Proença D Jr Bartholo R . VOSviewer and Bibliometrix. J Med Libr Assoc. (2022) 110:392–5. doi: 10.5195/jmla.2022.1434 PMC9782747 36589296

[25] Waltman L van Eck N Noyons . A unified approach to mapping and clustering of bibliometric networks. J Informetr. (2010) 4:629–35. doi: 10.1016/j.joi.2010.07.002

[26] van Eck NJ Waltman L . Software survey: VOSviewer, a computer program for bibliometric mapping. Scientometrics. (2010) 84:523–38. doi: 10.1007/s11192-009-0146-3 PMC2883932 20585380

[27] Massimo A Corrado C . Bibliometrix: An R-tool for comprehensive science mapping analysis. J Informetr. (2017) 11:959–75. doi: 10.1016/j.joi.2017.08.007

[28] Lu C Liu M Shang W Yuan Y Li M Deng X . Knowledge mapping of angelica sinensis (Oliv.) diels (Danggui) research: a scientometric study. Front Pharmacol. (2020) 11:294. doi: 10.3389/fphar.2020.00294 32231572 PMC7082756

[29] Darby SC Ewertz M McGale P Bennet AM Blom-Goldman U Brønnum D . Risk of ischemic heart disease in women after radiotherapy for breast cancer. N Engl J Med. (2013) 368:987–98. doi: 10.1056/NEJMoa1209825 23484825

[30] Piroth MD Baumann R Budach W Dunst J Feyer P Fietkau R . Heart toxicity from breast cancer radiotherapy : Current findings, assessment, and prevention. Strahlenther Onkol. (2019) 195:1–12. doi: 10.1007/s00066-018-1378-z PMC6329735 30310926

[31] Taunk NK Haffty BG Kostis JB Goyal S . Radiation-induced heart disease: pathologic abnormalities and putative mechanisms. Front Oncol. (2015) 5:39. doi: 10.3389/fonc.2015.00039 25741474 PMC4332338

[32] Atkins KM Rawal B Chaunzwa TL Lamba N Bitterman DS Williams CL . Cardiac radiation dose, cardiac disease, and mortality in patients with lung cancer. J Am Coll Cardiol. (2019) 73:2976–87. doi: 10.1016/j.jacc.2019.03.500 31196455

[33] Li H An H Wang Y Huang J Gao X . Evolutionary features of academic articles co-keyword network and keywords co-occurrence network: based on two-mode affiliation network. Physica a-Statistical Mechanics Its Appl. (2016) 450:657–69. doi: 10.1016/j.physa.2016.01.017

[34] Shi Y Wei W Li L Wei Q Jiang F Xia G . The global status of research in breast cancer liver metastasis: a bibliometric and visualized analysis. Bioengineered. (2021) 12:12246–62. doi: 10.1080/21655979 PMC8810156 34783637

[35] Kinsella TJ Ahmann DL Giuliani ER Lie JT . Adriamycin cardiotoxicity in stage IV breast cancer: possible enhancement with prior left chest radiation therapy. Int J Radiat Oncol Biol Phys. (1979) 5:1997–2002. doi: 10.1016/0360-3016(79)90951-9 397210

[36] Olive D . Cardiac toxicity of chemotherapy and radiation-therapy. Arch francaises pediatrie. (1981) 38:379–84. 7020629

[37] Baselet B Sonveaux P Baatout S Aerts A . Pathological effects of ionizing radiation: endothelial activation and dysfunction. Cell Mol Life Sci. (2019) 76:699–728. doi: 10.1007/s00018-018-2956-z 30377700 PMC6514067

[38] Ping Z Peng Y Lang H Xinyong C Zhiyi Z Xiaocheng W . Oxidative stress in radiation-induced cardiotoxicity. Oxid Med Cell Longev. (2020) 2020:3579143. doi: 10.1155/2020/3579143 32190171 PMC7071808

[39] Wang B Wang H Zhang M Ji R Wei J Xin Y . Radiation-induced myocardial fibrosis: Mechanisms underlying its pathogenesis and therapeutic strategies. J Cell Mol Med. (2020) 24:7717–29. doi: 10.1111/jcmm.15479 PMC7348163 32536032

[40] Banfill K Giuliani M Aznar M Franks K McWilliam A Schmitt M . Cardiac toxicity of thoracic radiotherapy: existing evidence and future directions. J Thorac Oncol. (2021) 16:216–27. doi: 10.1016/j.jtho.2020.11.002 PMC7870458 33278607

[41] Schlaak RA SenthilKumar G Boerma M Bergom C . Advances in preclinical research models of radiation-induced cardiac toxicity. Cancers. (2020) 12:415. doi: 10.3390/cancers12020415 32053873 PMC7072196

[42] Dabjan MB Buck CM Jackson IL Vujaskovic Z Marples B Down JD . A survey of changing trends in modelling radiation lung injury in mice: bringing out the good, the bad, and the uncertain. Lab Invest. (2016) 96:936–49. doi: 10.1038/labinvest.2016.76 27479087

[43] Ghita M Dunne V Hanna GG Prise KM Williams JP Butterworth KT . Preclinical models of radiation-induced lung damage: challenges and opportunities for small animal radiotherapy. Br J Radiol. (2019) 92:20180473. doi: 10.1259/bjr.20180473 30653332 PMC6541188

[44] Sharma S Moros EG Boerma M Sridharan V Han EY Clarkson R . A novel technique for image-guided local heart irradiation in the rat. Technol Cancer Res Treat. (2014) 13:593–603. doi: 10.7785/tcrtexpress.2013.600256 24000983 PMC3951712

[45] Loap P Fourquet A Kirova Y . Should we move beyond mean heart dose? Int J Radiat Oncol Biol Phys. (2020) 107:386–7. doi: 10.1016/j.ijrobp.2020.02.017 32386738

[46] Kasmann L Taugner J Manapov F . Chemo-/immuno-/radiotherapy combination in treatment of solid cancer. Oncotarget. (2019) 10:5387–8. doi: 10.18632/oncotarget.27141 PMC6739220 31534625

[47] van Nimwegen FA Schaapveld M Janus CP Krol AD Petersen EJ Raemaekers JM . Cardiovascular disease after Hodgkin lymphoma treatment: 40-year disease risk. JAMA Intern Med. (2015) 175:1007–17. doi: 10.1001/jamainternmed.2015.1180 25915855

[48] Pinder MC Duan Z Goodwin JS Hortobagyi GN Giordano SH . Congestive heart failure in older women treated with adjuvant anthracycline chemotherapy for breast cancer. J Clin Oncol. (2007) 25:3808–15. doi: 10.1200/JCO.2006.10.4976 17664460

[49] Fajardo LF Eltringham JR Steward JR . Combined cardiotoxicity of adriamycin and x-radiation. Lab Invest. (1976) 34:86–96. 1246126

[50] Dreyfuss AD Velalopoulou A Avgousti H Bell BI Verginadis II . Preclinical models of radiation-induced cardiac toxicity: Potential mechanisms and biomarkers. Front Oncol. (2022) 12:920867. doi: 10.3389/fonc.2022.920867 36313656 PMC9596809

[51] Boerma M . Experimental radiation-induced heart disease: past, present, and future. Radiat Res. (2012) 178:1–6. doi: 10.1667/RR2933.1 22663150 PMC3423081

[52] Pogwizd SM Bers DM . Rabbit models of heart disease. Drug Discovery Today Dis Models. (2008) 5:185–93. doi: 10.1016/j.ddmod.2009.02.001 PMC7105925 32288771

[53] Camacho P Fan H Liu Z He JQ . Large Mammalian animal models of heart disease. J Cardiovasc Dev Dis. (2016) 3:30. doi: 10.3390/jcdd3040030 29367573 PMC5715721

[54] van Nimwegen FA Schaapveld M Cutter DJ Janus CP Krol AD Hauptmann M . Radiation dose-response relationship for risk of coronary heart disease in survivors of Hodgkin lymphoma. J Clin Oncol. (2016) 34:235–43. doi: 10.1200/JCO.2015.63.444 26573075

[55] Sardar P Kundu A Chatterjee S Nohria A Nairooz R Bangalore S . Long-term cardiovascular mortality after radiotherapy for breast cancer: A systematic review and meta-analysis. Clin Cardiol. (2017) 40:73–81. doi: 10.1002/clc.22631 28244595 PMC6490535

[56] Bradley JD Paulus R Komaki R Masters G Blumenschein G Schild S . Standard-dose versus high-dose conformal radiotherapy with concurrent and consolidation carboplatin plus paclitaxel with or without cetuximab for patients with stage IIIA or IIIB non-small-cell lung cancer (RTOG 0617): a randomised, two-by-two factorial phase 3 study. Lancet Oncol. (2015) 16:187–99. doi: 10.1016/S1470-2045(14)71207-0 PMC4419359 25601342

[57] Speirs CK DeWees TA Rehman S Molotievschi A Velez MA Mullen D . Heart dose is an independent dosimetric predictor of overall survival in locally advanced non-small cell lung cancer. J Thorac Oncol. (2017) 12:293–301. doi: 10.1016/j.jtho.2016.09.134 27743888

[58] Shah AB Hudson MM Poquette CA Luo X Wilimas JA Kun LE . Long-term follow-up of patients treated with primary radiotherapy for supradiaphragmatic Hodgkin's disease at St. Jude Children's Res Hospital Int J Radiat Oncol Biol Phys. (1999) 44:867–77. doi: 10.1016/s0360-3016(99)00052-8 10386644

[59] Specht L Yahalom J Illidge T Berthelsen AK Constine LS Eich HT . Modern radiation therapy for Hodgkin lymphoma: field and dose guidelines from the International Lymphoma Radiation Oncology Group (ILROG). Int J Radiat Oncol Biol Phys. (2014) 89:854–62. doi: 10.1016/j.ijrobp.2013.05.005 23790512

[60] Wirth A Mikhaeel NG Aleman BMP Pinnix CC Constine LS Ricardi U . Involved site radiation therapy in adult lymphomas: an overview of International Lymphoma Radiation Oncology Group guidelines. Int J Radiat Oncol Biol Phys. (2020) 107:909–33. doi: 10.1016/j.ijrobp.2020.03.019 32272184

[61] Mehta LS Watson KE Barac A Beckie TM Bittner V Cruz-Flores S . Cardiovascular disease and breast cancer: where these entities intersect: a scientific statementfrom the American Heart Association. Circulation. (2018) 137:e30–66. doi: 10.1161/CIR.0000000000000556 PMC6722327 29437116

[62] Aleman BM van den Belt-Dusebout AW De Bruin ML van 't Veer MB Baaijens MH de Boer JP . Late cardiotoxicity after treatment for Hodgkin lymphoma. Blood. (2007) 109:1878–86. doi: 10.1182/blood-2006-07-034405 17119114

[63] Bergom C Bradley JA Ng AK Samson P Robinson C Lopez-Mattei J . Past, present, and future of radiation-induced cardiotoxicity: refinements in targeting, surveillance, and risk stratification. JACC CardioOncol. (2021) 3:343–59. doi: 10.1016/j.jaccao.2021.06.007 PMC8463722 34604796

[64] Mulrooney DA Yeazel MW Kawashima T Mertens AC Mitby P Stovall M . Cardiac outcomes in a cohort of adult survivors of childhood and adolescent cancer: retrospective analysis of the Childhood Cancer Survivor Study cohort. BMJ. (2009) 339:b4606. doi: 10.1136/bmj.b4606 19996459 PMC3266843

[65] Aleman BM van den Belt-Dusebout AW De Bruin ML van 't Veer MB Baaijens MH de Boer JP . Valvular dysfunction and carotid, subclavian, and coronary artery disease in survivors of hodgkin lymphoma treated with radiation therapy. JAMA. (2003) 290:2831–7. doi: 10.1001/jama.290.21.2831 14657067

[66] Taylor CW Nisbet A McGale P Darby SC . Cardiac exposures in breast cancer radiotherapy: 1950s-1990s. Int J Radiat Oncol Biol Phys. (2007) 69:1484–95. doi: 10.1016/j.ijrobp.2007.05.034 18035211

[67] Polonsky TS McClelland RL Jorgensen NW Bild DE Burke GL Guerci AD . Coronary artery calcium score and risk classification for coronary heart disease prediction. JAMA. (2010) 303:1610–6. doi: 10.1001/jama.2010.461 PMC3033741 20424251

[68] Jordan JH Todd RM Vasu S Hundley WG . Cardiovascular magnetic resonance in the oncology patient. JACC Cardiovasc Imaging. (2018) 11:1150–72. doi: 10.1016/j.jcmg.2018.06.004 PMC6242266 30092971

[69] Demissei BG Freedman G Feigenberg SJ Plastaras JP Maity A Smith AM . Early changes in cardiovascular biomarkers with contemporary thoracic radiation therapy for breast cancer, lung cancer, and lymphoma. Int J Radiat Oncol Biol Phys. (2019) 103:851–60. doi: 10.1016/j.ijrobp.2018.11.013 PMC6722323 30445173

[70] Dreyfuss AD Goia D Shoniyozov K Shewale SV Velalopoulou A Mazzoni S . A novel mouse model of radiation-induced cardiac injury reveals biological and radiological biomarkers of cardiac dysfunction with potential clinical relevance. Clin Cancer Res. (2021) 27:2266–76. doi: 10.1158/1078-0432.CCR-20-3882 PMC9215266 33542079

[71] Ell P Martin JM Cehic DA Ngo DTM Sverdlov AL . Cardiotoxicity of radiation therapy: mechanisms, management, and mitigation. Curr Treat Options Oncol. (2021) 22:70. doi: 10.1007/s11864-021-00868-7 34110500

[72] Giordano SH Kuo YF Freeman JL Buchholz TA Hortobagyi GN Goodwin JS . Risk of cardiac death after adjuvant radiotherapy for breast cancer. J Natl Cancer Inst. (2005) 97:419–24. doi: 10.1093/jnci/dji067 PMC1853253 15770005

[73] Reardon KA Read PW Morris MM Reardon MA Geesey C Wijesooriya K . A comparative analysis of 3D conformal deep inspiratory-breath hold and free-breathing intensity-modulated radiation therapy for left-sided breast cancer. Med Dosim. (2013) 38:190–5. doi: 10.1016/j.meddos.2013.01.002 23453454

[74] Berg M Lorenzen EL Jensen I Thomsen MS Lutz CM Refsgaard L . The potential benefits from respiratory gating for breast cancer patients regarding target coverage and dose to organs at risk when applying strict dose limits to the heart: results from the DBCG HYPO trial. Acta Oncol. (2018) 57:113–9. doi: 10.1080/0284186X.2017.1406139 29205080

[75] Lymberis SC deWyngaert JK Parhar P Chhabra AM Fenton-Kerimian M Chang J . Prospective assessment of optimal individual position (prone versus supine) for breast radiotherapy: volumetric and dosimetric correlations in 100 patients. Int J Radiat Oncol Biol Phys. (2012) 84:902–9. doi: 10.1016/j.ijrobp.2012.01.040 22494590

[76] McDonald MW Godette KD Butker EK Davis LW Johnstone PA . Long-term outcomes of IMRT for breast cancer: a single-institution cohort analysis. Int J Radiat Oncol Biol Phys. (2008) 72:1031–40. doi: 10.1016/j.ijrobp.2008.02.053 18440727

[77] Lazzari G Terlizzi A Leo MG Silvano G . VMAT radiation-induced nausea and vomiting in adjuvant breast cancer radiotherapy: the incidental effect of lowdose bath exposure. Clin Translat Radiat Oncol. (2017) 7:43–8. doi: 10.1016/j.ctro.2017.09.009 PMC5862677 29594228

[78] Frankart AJ Nagarajan R Pater L . The impact of proton therapy on cardiotoxicity following radiation treatment. J Thromb Thrombolysis. (2021) 51:877–83. doi: 10.1007/s11239-020-02303-4 33033980

[79] Vijayakumar S Chen GT . Implementation of three dimensional conformal radiation therapy: prospects, opportunities, and challenges. Int J Radiat Oncol Biol Phys. (1995) 33:979–83. doi: 10.1016/0360-3016(95)02060-8 7493859

[80] Chun SG Hu C Choy H Komaki RU Timmerman RD Schild SE . Impact of intensity-modulated radiation therapy technique for locally advanced non-small-cell lung cancer: A secondary analysis of the NRG oncology RTOG 0617 randomized clinical trial. J Clin Oncol. (2017) 35:56–62. doi: 10.1200/JCO.2016.69.1378 28034064 PMC5455690

[81] Murakami YM Kamima T Abo N Takahashi T Kaneko M Nakano M . Dosimetric comparison between 3D conformal radiation therapy plus electron boost and simultaneous integrated boost volumetric modulated arc therapy for left-sided breast cancer patients with a potential risk of radiation-induced cardiac toxicity. Int J Radiat Oncol Biol Phys. (2021) 111:525–6. doi: 10.1016/j.ijrobp.2021.07.1435

[82] LaRiviere MJ Santos PMG Hill-Kayser CE Metz JM . Proton therapy. Hematol Oncol Clin North Am. (2019) 33:989–1009. doi: 10.1016/j.hoc.2019.08.006 31668216

[83] Lin SH Hobbs BP Verma V Tidwell RS Smith GL Lei X . Randomized phase IIB trial of proton beam therapy versus intensity-modulated radiation therapy for locally advanced esophageal cancer. J Clin Oncol. (2020) 38:1569–79. doi: 10.1200/JCO.19.02503 PMC7213588 32160096

[84] Fritz G Henninger C Huelsenbeck J . Potential use of HMG-CoA reductase inhibitors (statins) as radioprotective agents. Br Med Bull. (2011) 97:17–26. doi: 10.1093/bmb/ldq044 21252099

[85] Camara Planek MI Silver AJ Volgman AS Okwuosa TM . Exploratory review of the role of statins, colchicine, and aspirin for the prevention of radiation-associated cardiovascular disease and mortality. J Am Heart Assoc. (2020) 9:e014668. doi: 10.1161/JAHA.119.014668 31960749 PMC7033839

[86] Nestle U De Ruysscher D Ricardi U Geets X Belderbos J Pöttgen C . ESTRO ACROP guidelines for target volume definition in the treatment of locally advanced non-small cell lung cancer. Radiother Oncol. (2018) 127:1–5. doi: 10.1016/j.radonc.2018.02.023 29605476

[87] Duane F Aznar MC Bartlett F Cutter DJ Darby SC Jagsi R . A cardiac contouring atlas for radiotherapy. Radiother Oncol. (2017) 122:416–22. doi: 10.1016/j.radonc.2017.01.008 PMC5356506 28233564

[88] Feng M Moran JM Koelling T Chughtai A Chan JL Freedman L . Development and validation of a heart atlas to study cardiac exposure to radiation following treatment for breast cancer. Int J Radiat Oncol Biol Phys. (2011) 79:10–8. doi: 10.1016/j.ijrobp.2009.10.058 PMC2937165 20421148

[89] Kong FM Ritter T Quint DJ Senan S Gaspar LE Komaki RU . Consideration of dose limits for organs at risk of thoracic radiotherapy: atlas for lung, proximal bronchial tree, esophagus, spinal cord, ribs, and brachial plexus. Int J Radiat Oncol Biol Phys. (2011) 81:1442–57. doi: 10.1016/j.ijrobp.2010.07.1977 PMC3933280 20934273

[90] Bouleftour W Mery B Rowinski E Rivier C Daguenet E Magne N . Cardio-oncology preclinical models: A comprehensive review. Anticancer Res. (2021) 41:5355–64. doi: 10.21873/anticanres.15348 34732405

[91] Yusuf SW Venkatesulu BP Mahadevan LS Krishnan S . Radiation-induced cardiovascular disease: A clinical perspective. Front Cardiovasc Med. (2017) 4:66. doi: 10.3389/fcvm.2017.00066 29124057 PMC5662579

[92] Lancellotti P Nkomo VT Badano LP Bergler-Klein J Bogaert J Davin L . Expert consensus for multi-modality imaging evaluation of cardiovascular complications of radiotherapy in adults: a report from the European Association of Cardiovascular Imaging and the American Society of Echocardiography. J Am Soc Echocardiogr. (2013) 26:1013–32. doi: 10.1016/j.echo.2013.07.005 23998694

[93] Plana JC Galderisi M Barac A Ewer MS Ky B Scherrer-Crosbie M . Expert consensus for multimodality imaging evaluation of adult patients during and after cancer therapy: a report from the American Society of Echocardiography and the European Association of Cardiovascular Imaging. J Am Soc Echocardiogr. (2014) 27:911–39. doi: 10.1016/j.echo.2014.07.012 25172399

[94] McDonagh TA Blue L Clark AL Dahlström U Ekman I Lainscak M . European Society of Cardiology Heart Failure Association Standards for delivering heart failure care. Eur J Heart Fail. (2011) 13:235–41. doi: 10.1093/eurjhf/hfq22 21159794

